# The neuroendocrine puzzle of epilepsy and infertility: what are we missing?

**DOI:** 10.3389/fneur.2025.1658284

**Published:** 2025-11-13

**Authors:** Dominik Kobylarek, Katarzyna Zakryś, Julia Gierszewska, Bhavana Reddy Pagidela, Yung-Yi Lan, Rujith Kovinthapillai, Aleksander Rajczewski, Wojciech Kozubski, Sławomir Michalak

**Affiliations:** 1 Institute of Neurological Disorders, Poznan University of Medical Sciences, Poznań, Poland; 2 Poznan University of Medical Sciences, Doctoral School, Department of Neurology, Poznań, Poland

**Keywords:** epilepsy, infertility, hormonal dysregulation, sex hormone regulation, endocrine signaling pathways, fertility, pregnancy, fetal development

## Abstract

Epilepsy, a chronic neurological condition affecting over 70 million individuals worldwide, has far-reaching effects beyond seizure activity, including a significant impact on reproductive health by posing significant challenges to fertility and hormonal health. Emerging evidence underscores the complex bidirectional interplay between epilepsy, antiepileptic drugs (AEDs), and sex steroid hormones; particularly progesterone, testosterone, and prolactin, which influence both seizure threshold and reproductive function. This paper explores how epilepsy alters hypothalamic–pituitary-gonadal (HPG) axis dynamics, often leading to conditions like polycystic ovary syndrome (PCOS), anovulation, and menstrual irregularities. Moreover, the role of prolactin dysregulation following seizures, as well as the impact of temporal lobe epilepsy on gonadotropin-releasing hormone (GnRH) pulsatility, is examined in relation to infertility outcomes. By integrating current research on neuroendocrine signaling and reproductive physiology, this paper highlights the need for individualized care strategies in individuals with epilepsy to optimize both seizure control and reproductive health.

## Introduction

There is a wealth of evidence in the scientific literature supporting the complex relationship between epilepsy and infertility. Infertility is clinically defined as the failure to achieve pregnancy after 12 months of regular unprotected intercourse or as a result of reproductive disorders in one or both partners. Its prevalence is approximately 15% of couples worldwide (1, 2). The etiology of infertility is attributed to male factors in about one-third of cases, female factors in another third, and the remaining cases are considered idiopathic (3, 4).

Epilepsy is one of the most common neurological disorders, affecting over 70 million individuals worldwide (5, 6). Its development and manifestations are multifactorial and often reflect an interplay of genetics, environmental and acquired factors, and hormonal dysregulation (7, 8). Due to its mechanisms of neuronal disruption, epilepsy has a profound systemic impact and can significantly affect reproductive health through disruption of the hypothalamic–pituitary-gonadal (HPG) axis and alterations in circulating sex hormone levels (9, 10).

Women with epilepsy (WWE) commonly exhibit reproductive disturbances, including menstrual irregularities, polycystic ovary syndrome (PCOS), and dysregulation of estrogen, androgen, and prolactin (PRL) levels (11–13). Men with epilepsy (MWE) often present with hypogonadism, reduced testosterone, and impaired spermatogenesis (14). Disruption of the HPG axis, ion channel dysfunction, and anti-seizure medications (ASM)-mediated endocrine interference underlie these impairments (11, 15, 16). Key fertility regulators that are affected include estrogen, progesterone (PROG), prolactin (PRL), follicle-stimulating hormone (FSH), luteinizing hormone (LH), oxytocin (OT), dehydroepiandrosterone (DHEA), human chorionic gonadotropin (hCG), anti-müllerian hormone (AMH), testosterone, neurosteroids, and insulin (13–15). Despite significant advances in understanding the intersection between epilepsy and infertility, critical gaps remain regarding the mechanisms through which epilepsy and its treatment influence reproductive health. Key unanswered questions include the pathophysiological mechanisms underlying infertility in patients with epilepsy and the effective strategies for preserving fertility in affected individuals to improve quality of life both physiologically and psychosocially.

This review aims to explore the relationship between epilepsy and hormonal dysregulation, particularly emphasizing reproductive outcomes. It consolidates the current evidence regarding the complex interactions among epilepsy, sex hormone regulation, endocrine signaling pathways, and fertility. Additionally, it provides a comprehensive overview of how epilepsy impacts fertility, pregnancy, and fetal development, while considering the 2025 ILAE framework to address previous limitations that have hindered translational research. Furthermore, it discusses emerging strategies aimed at preserving reproductive function within this vulnerable population.

## The interplay between reproductive hormones and neuronal activity

The impact of reproductive hormones on seizure occurrence is well documented, especially during physiologic states of high hormonal influence such as menstruation, pregnancy, and menopause. Hormonal fluctuations can alter seizure control due to changes in neuronal excitability and survival, but strangely, pregnancy, a state of high hormonal influence, is not reported to exacerbate seizures (17, 18). Two-thirds of women remain free from seizures during pregnancy, although if seizures are present, there may be a significant worsening of the general condition, especially due to focal seizures. In such cases, close supervision of the treatment regimen and seizure response is required, with modifications such as increased dosages of ASMs or different drug options (17, 18).

Women with epilepsy (WWE) are significantly more likely to experience reproductive and sexual dysfunction than the general population. Sexual disorders affect approximately 20–30% of WWE, with reduced libido being the most common complaint (19). Additional endocrine and reproductive abnormalities frequently reported include polycystic ovary syndrome (PCOS), menstrual irregularities, premature menopause, hyperandrogenism, hypogonadotropic hypogonadism, premature ovarian failure, and hyperprolactinemia (20–26). Approximately 33% of WWE experience seizure exacerbation corresponding with the changes of the menstrual cycle, known as catamenial epilepsy (27). This seizure pattern is sensitive to changes in hormonal levels of estrogen and progesterone (PROG), both key players in modulating neuronal excitation and thereby seizure thresholds (28). Corresponding to the dominant hormone in each phase of the menstrual cycle, three main patterns of catamenial epilepsy have been noted. The C1 (perimenstrual pattern) is guided by a decline in PROG and its metabolites, which reduces GABAergic inhibition, consequently increasing seizure susceptibility. The C2 (perimenstrual pattern) is characterized by a peak in E2 (estradiol) levels, which enhances the excitation of neurons, and lastly, the C3 (anovulatory pattern) is marked by a highly sustained level of estrogen in the absence of PROG entirely, leading to heightened neuronal excitation (29–31). These disruptions in hormonal balance not only reflect underlying endocrine abnormalities but also contribute to reduced fertility. Morrell estimates that 50–66% of WWE may be at risk for impaired fertility compared to women without epilepsy (32).

The hormonal effects of epilepsy are not limited to women, but are highly reported in men as well, with rates of sexual dysfunction ranging between 38 to 71% among men with epilepsy (MWE) (33). Hormonal dysregulation caused by epilepsy results in reduced levels of testosterone, both in its free and albumin-bound state, lower free androgen index (FAI) and increased concentrations of estradiol (E2), sex hormone-binding globulin (SHBG), luteinizing hormone (LH), follicle-stimulating hormone (FSH), and prolactin (PRL) (22, 23, 34). This imbalance and marked decrease in testosterone concentrations manifests as concerns such as delayed sexual development, impaired spermatogenesis, decreased libido, and abnormal testicular morphology (20, 22). MWE have characteristically lower levels of testosterone, even from younger ages, where 11% of individuals under 20 years of age exhibit sub-threshold levels. These manifestations become more pronounced with age as testosterone levels decrease physiologically as well, causing increased sexual dysfunction in MWE (35).

Although both sexes experience epilepsy-related endocrine disturbances, the clinical manifestations differ. These sex-specific factors warrant the need for personalized, comprehensive care that addresses both the neurological and psychosocial challenges associated with epilepsy (3, 11, 12, 34).

## The mechanisms of hormonal disturbances caused by epilepsy

The relationship between epilepsy and sex hormones is complex and not yet fully understood and has a multi-faceted interaction since both the condition itself and the use of ASMs can disrupt the hypothalamic–pituitary axis, resulting in abnormal steroid hormone production (20). One theory proposes that alterations in neuronal activity during and between seizures interrupt the release of neurotransmitters glutamate, NE, GABA, and dopamine which interferes with the pulsatility of Gonadotropin-releasing Hormone (GnRH) and consequent hormonal regulation (20, 61). It has been suggested that epilepsy directly affects the hypothalamic–pituitary axis, resulting in altered levels of GnRH, LH, FSH, PRL, and downstream hormones such as estrogen, testosterone, and DHEA (20).

GnRH pulse frequency plays a critical role in determining gonadotropin output. Low-frequency pulses favor FSH secretion, while high-frequency pulses promote LH release. Continuous (non-pulsatile) GnRH secretion suppresses both FSH and LH, inhibiting ovulation and estrogen production. Seizure-related disruptions in this balance can thus result in reproductive dysfunction (64). Abnormal GnRH pulsatility is pathognomonic for several reproductive disorders. In PCOS, persistently high-frequency GnRH pulses shift the LH/FSH ratio toward LH dominance (65). This stimulates ovarian theca cells to overproduce androgens, while insufficient FSH impairs aromatase activity in granulosa cells, reducing estrogen synthesis. Conversely, in hypogonadotropic hypogonadism and hypothalamic amenorrhea, GnRH secretion is diminished, resulting in low FSH and LH levels and impaired gonadal function (66).

The pattern and location of seizures affect the prevalence of specific hormonal disturbances. This suggests that there is a direct link between epilepsy and endocrine dysfunction (21, 37–42). Present research is largely acquired by conducting investigations in animal models utilising electrical stimulation, and human studies using electroconvulsive therapy. Both models have demonstrated altered pituitary hormone secretion caused by altered neuron firing in response to epilepsy (43, 44). Hormonal changes have been noted both in response to seizures and during interictal periods, especially with increased levels of PRL and LH in both sexes (20, 37).

Dana-Haeri et al. (39) found that PRL and LH levels increased significantly 20 min after the seizure. Following a 60-min period, PRL returned to its initial level, while LH continued to demonstrate elevated levels for a longer duration of time. FSH was also noted to be increased both immediately after a seizure and following a 60-min period, but only in women. In a similar vein, Nappi et al. (45) reported a higher frequency of LH surges in both focal and generalised epilepsy cases. Elevated PRL levels may cause decreased libido and impotence in men, and anovulatory cycles in women (46, 47).

**Figure 1:**
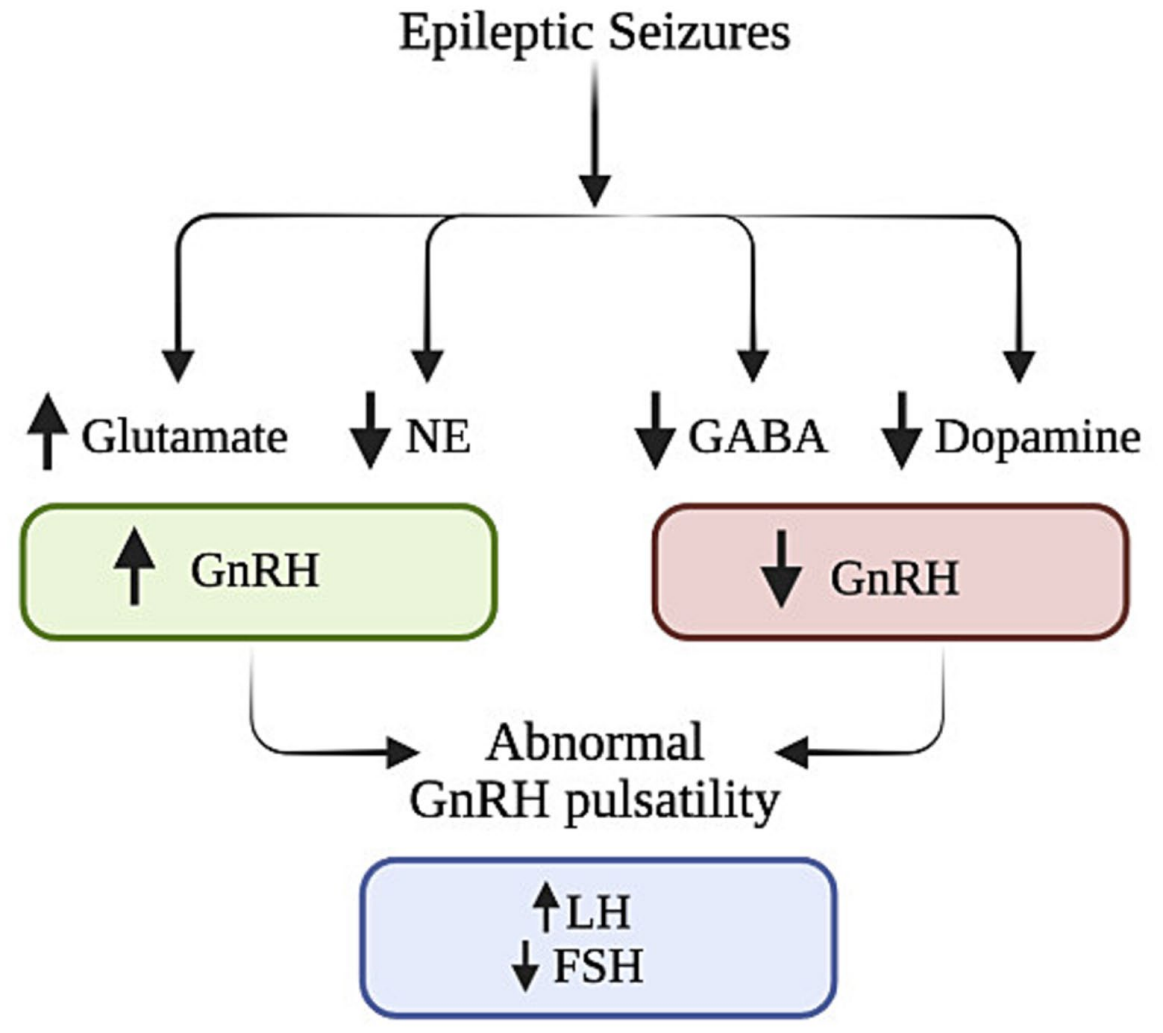
Neurotransmitters (glutamate, NE, GABA, dopamine) and hormonal pathways (GnRH, LH, FSH) involved in epileptic seizures, highlighting abnormal GnRH pulsatility as a potential factor in seizure-related endocrine disruptors. Created in BioRender (2025).

**Figure 2:**
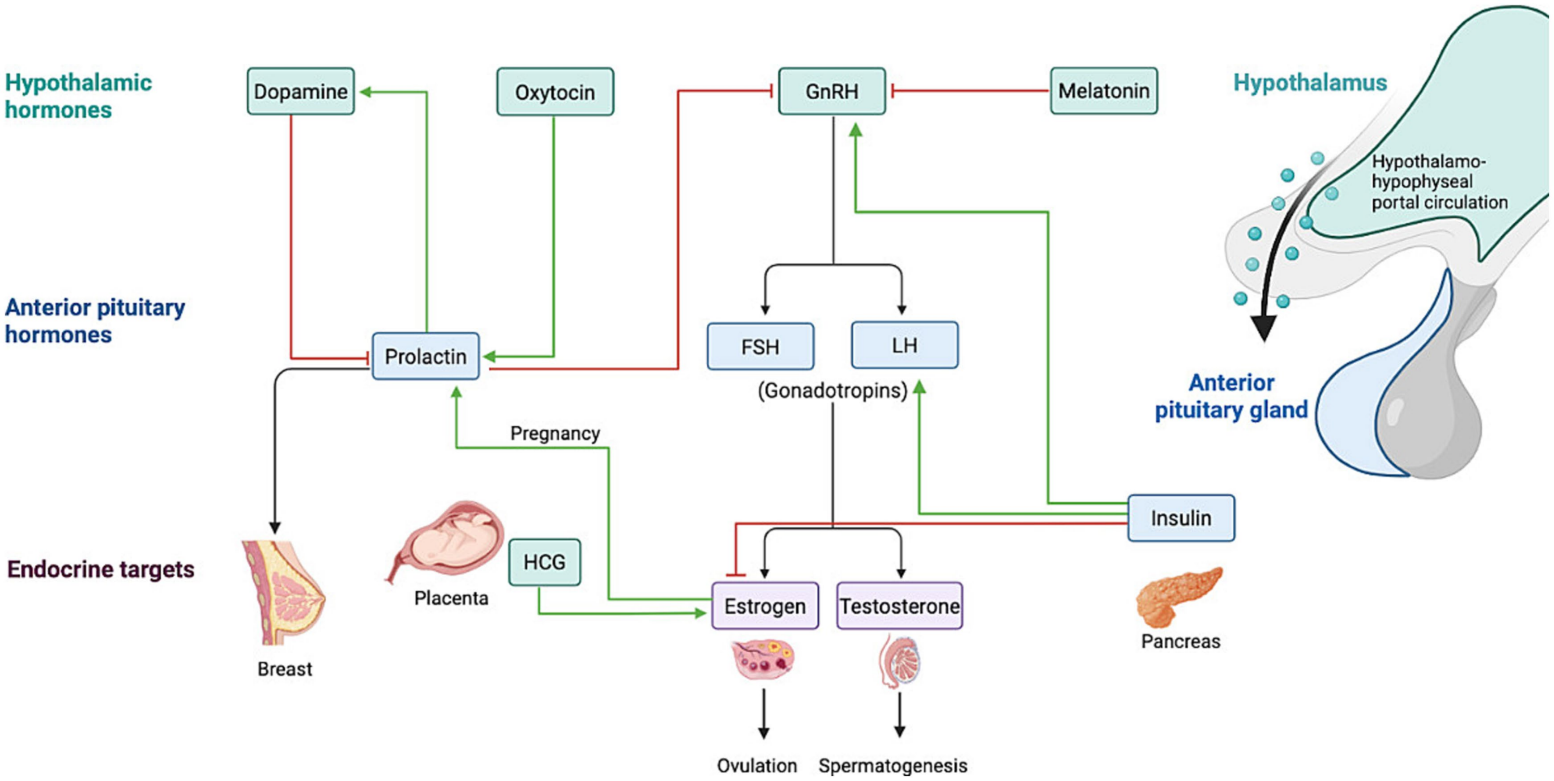
An overview of hypothalamic and anterior pituitary hormones, their endocrine targets, and key physiological processes such as ovulation, spermatogenesis, and pregnancy. The image highlights the roles of dopamine, oxytocin, GnRH, prolactin, FSH, LH, and other hormones in regulating bodily functions. Created in BioRender (2025).

**Figure 3:**
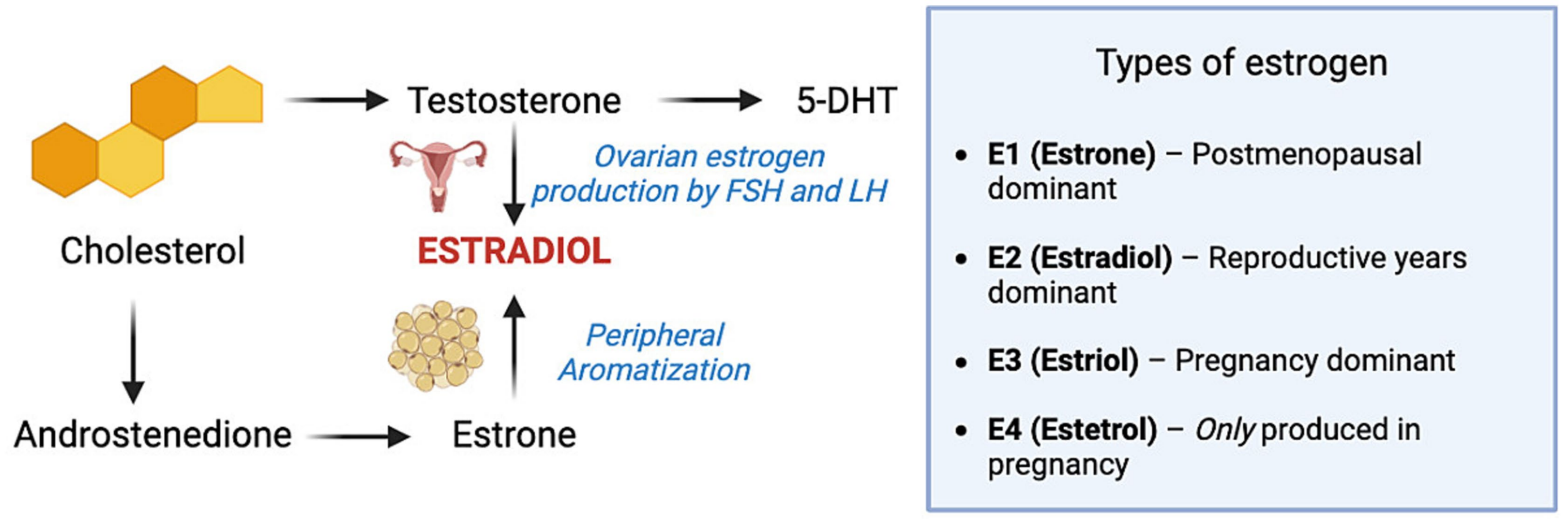
Metabolic pathways of androgen and estrogen biosynthesis, including cholesterol-derived intermediates and the roles of estrogen isoforms (E1–E4) across various reproductive stages. Created in BioRender (2025).

**Figure 4:**
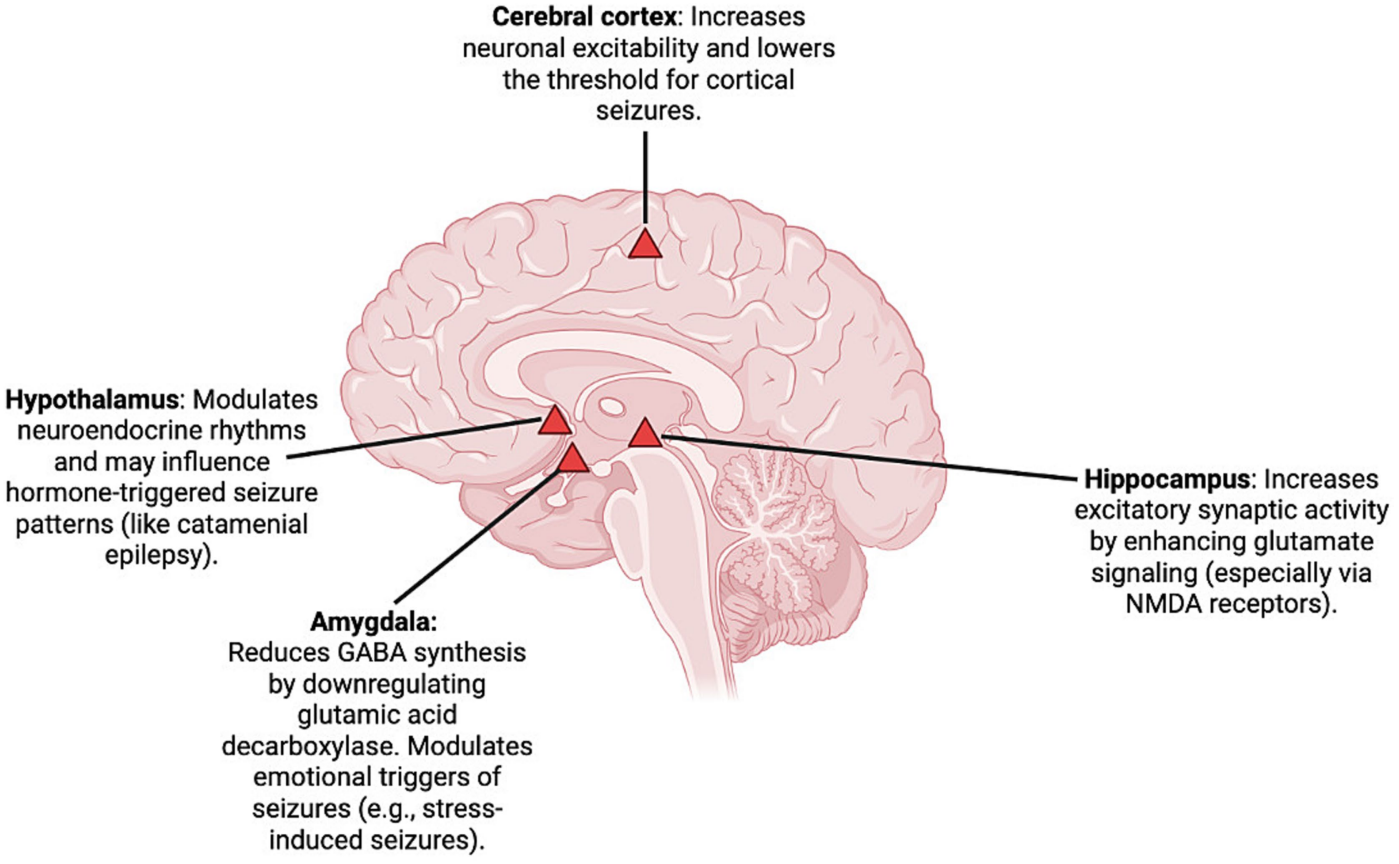
Brain regions with estrogen receptors and their roles in seizure susceptibility. Estrogen increases neuronal excitability in the cerebral cortex (promoting generalized seizures), modulates hormone-sensitive rhythms in the hypothalamus (linked to catamenial epilepsy), reduces GABAergic inhibition in the amygdala (enhancing emotional triggers), and enhances glutamate signaling in the hippocampus (a key focus in temporal lobe epilepsy). These mechanisms collectively lower the seizure threshold under hormonal fluctuations. Created in BioRender (2025).

Beyond the disease itself, ASMs also contribute to GnRH dysregulation by modulating neurotransmitters such as gamma-aminobutyric acid (GABA), NMDA, and glutamate in the limbic system (50). Epilepsy impacts hormonal regulation through feedback loops involving the hypothalamus, pituitary gland, and peripheral endocrine organs. Disruptions in these circuits, whether during seizures or interictal periods, impairs hormonal balance and regulation (51) (Figures 1–4).

## FSH, LH, and the HPG axis in epilepsy

Seizures affect the HPG axis which mainly regulate the release of sexual hormones. The downstream effect of the preoptic nucleus neurons activation in the hypothalamus result from the secretion of GnRH into the portal circulation and anterior pituitary gland activation (55). GnRH signal transduction to G-protein coupled receptors on the gonadotroph cells, generates LH and FSH release in the anterior pituitary (56, 57). Both hormones bind to receptors in the gonads and promote the production of sex hormones such as estrogen (E2), testosterone, and PROG (52, 58). Not only are these steroid hormones and their neuroactive metabolites highly influenced by seizures, they also modulate neuronal excitability through genomic and non-genomic mechanisms, thus influencing seizure susceptibility (18, 59). The bidirectional impact of endocrine dysregulation causes higher levels of hormonal and reproductive disruption in individuals with epilepsy when compared to their healthy counterparts, in both men and women (20, 53, 54). The dysfunction is not limited to any subtype of epilepsy, but is rather observed in the vast majority of cases, including focal and generalized onset seizures as well as secondary generalized focal onset seizures (20) but is most commonly associated with temporal lobe epilepsy (TLE) (53, 54).

TLE, a specific subtype of focal epilepsy affecting mainly the temporal lobe, exerts a more profound effect on the HPG axis, leading to significant dysregulation of gonadotropin secretion, especially impacting FSH and LH (8). TLE can trigger seizure activity in different hemispheres, each exerting lateralized effects on neuroendocrine regulation. Left-sided TLE is consistently associated with elevated GnRH pulse frequency, triggering higher LH/FSH ratios resulting in hyperandrogenism, and clinical features resembling PCOS (8). In contrast, right-sided TLE is more often linked to hypogonadotropic hypogonadism, characterized by reduced LH and FSH secretion resulting in amenorrhea (48). Essentially, left temporal foci contribute to hypergonadotropic states, whereas right temporal foci contribute to hypogonadism. In addition to the chronic region-specific impact of epilepsy on the HPG axis, acute ictal and postictal events also produce immediate hormonal disturbances. Postictal surges of neuronal activity trigger surges of LH and FSH translating immediately to short-term endocrine imbalances. Chronic instability of these hormones often terminates in altered LH pulse frequency and amplitude, causing anovulatory cycles and menstrual irregularities (49).

Up to 50% of WWE experience reproductive disturbances such as oligomenorrhea, amenorrhea, and infertility. These effects are more pronounced in women with left-sided epileptiform discharges, which may more directly impact limbic-hypothalamic pathways responsible for GnRH regulation (63).

Seizure disorders and ASMs, particularly valproic acid (VPA), can further exacerbate GnRH dysregulation. VPA has been shown to elevate androgen levels and worsen LH/FSH imbalances, contributing to PCOS-like features in women and hormonal suppression in men (15, 42, 48, 62, 68).

In MWE, elevated LH levels may reflect increased pituitary drive in response to testicular dysfunction. Increased FSH and LH levels are markers of gonadal damage and impaired spermatogenesis. Epileptic discharges may interfere with hypothalamic control of testosterone production via Leydig cell suppression and disruption of negative feedback loops. Clinically, this manifests as reduced libido, delayed sexual development, and infertility (69).

The clinical consequences of HPG axis disruption in epilepsy are extensive. Menstrual irregularities, infertility, and premature menopause are observed frequently in women, especially those with TLE and VPA exposure. Men are similarly affected, with hypogonadism and sexual dysfunction being the typical manifestations of reproductive disruption. These findings emphasize the importance of integrated treatment strategies that address seizure control and reproductive health.

Seizure activity during ictal and postictal phases can interfere with the pulsatile release of GnRH, likely due to disrupted neurotransmitter signaling involving GABA, glutamate, and NMDA receptor pathways (60). Ictal events in TLE have been shown to trigger transient postictal surges in both LH and FSH, leading to acute hormonal fluctuations (49). This disruption contributes to altered levels of LH, FSH, PRL, estrogen, and testosterone (20, 61).

Generalized seizures are also associated with acute elevations in PRL and LH in both sexes, while FSH levels tend to rise predominantly in women. PRL typically returns to baseline within an hour, but LH may remain elevated for longer periods (37, 62). Chronic seizure activity can lead to persistent alterations in the frequency and amplitude of LH pulses, often resulting in anovulatory cycles and menstrual dysfunction.

## Estrogen

Estrogens are a group of sex steroid hormones that include estrone (E1), estradiol (E2), estriol (E3), and estetrol (E4), the latter being produced exclusively during pregnancy. These hormones are synthesized from cholesterol, the main precursor to all steroid hormones, including testosterone and androstenedione, which are immediate precursors to estrogen, via the enzyme aromatase. The biosynthesis is tightly controlled by alterations in LH and FSH levels, secreted by the anterior pituitary in response to GnRH (70). The production of estrogens in women occurs primarily in the ovaries, while in men, 20% of estrogen is derived from the testes but predominantly produced through peripheral aromatization of adipose tissue, bone, brain, and skin (71).

Based on the physiological state of the body, the prevailing estrogen varies. E2 is the main estrogen during reproductive years, E1 dominates after menopause, and E3 becomes prominent during pregnancy (72–74). While estrogen is highlighted as the main hormone regulating female reproductive physiology, its importance in males is often overlooked but very critical. Inadequate estrogen signaling in the testes can impair spermatogenesis and contribute to infertility (70).

Beyond their reproductive roles, estrogens exert a significant influence on brain function. These hormones are locally synthesized in the brain via aromatization and play a key role in modulating cognitive and neuroendocrine process in key regions of the brain especially the hypothalamus and temporolimbic system, including the medial and cortical amygdala (75, 76). In these regions, estrogens highly influence emotional regulation, fear and anxiety processing as well as neuronal excitability via estrogen receptors ESR1, ESR2, and G-protein coupled estrogen receptor (GPER) (70, 77). E2 in particular is implicated in altering seizure susceptibility in regions such as the hippocampus, cerebral cortex, amygdala, and hypothalamus by altering key neurotransmitters such as GABA and glutamate (77).

Historically, estrogens were believed to be purely proconvulsant based on early animal and clinical data. However, accumulating evidence suggests that estrogens can exhibit both proconvulsant and anticonvulsant properties, with their effects modulated by factors such as sex, hormonal status, seizure type, brain region, dosage, and duration of exposure (78, 79). This duality as well as the influence of various external factors, complicates predictions of their influence on seizure activity.

At a cellular level, E2 enhances excitatory neurotransmission by stimulating NMDA receptor-mediated glutamate activity, thereby increasing neuronal firing rates (80). It augments the excitability of hippocampal CA1 pyramidal neurons and weakens inhibitory control by reducing GABA synthesis and downregulating glutamic acid decarboxylase in the corticomedial amygdala (76, 81). E2 also elevates acetylcholine levels and increases choline acetyltransferase activity, further potentiating excitatory activity (82).

Conversely, E2 has demonstrated anticonvulsant and neuroprotective effects in several preclinical studies. Due to its ability to counteract cyclosporin A-induced inhibition of GABAergic signaling in the hippocampus, estrogen thereby reduces seizure susceptibility (84). In ovariectomized female rats, estrogen administration attenuated seizures induced by NMDA, kainic acid (KA), cyclosporin A, and picrotoxin, supporting its role in enhancing GABAergic tone and modulating excitatory input (77, 83–86). Additionally, E1 reduced KA-induced seizures and mortality in male mice (87), and E2 pre-treatment in female rats protected against hippocampal damage following status epilepticus (SE) (88, 89).

Clinically, estrogen imbalance has profound consequences for individuals with epilepsy, particularly during states such as pregnancy or menopause, where hormonal regulation is strictly controlled and plays a key role in physiological processes. Variations in E1, E2, and E3 levels can modulate neuronal excitability and thus affects seizure thresholds. Creating a feedback loop and hormonal fluctuations cause bidirectional influence. Moreover, hormone replacement therapy (HRT) and certain contraceptives may influence seizure patterns, depending on their formulation and estrogenic content (90).

## Progesterone

Progesterone (PROG) is a key sex steroid hormone involved in reproductive and neuroendocrine function. In females, it is primarily secreted by the corpus luteum during the luteal phase of the menstrual cycle and in early pregnancy, while placental production dominates from the second trimester onward. PROG facilitates implantation, supports gestation, and contributes to maintaining pregnancy. In males, smaller amounts of PROG are synthesized by the testes and adrenal glands, where it plays critical roles in spermatogenesis, sexual function, and neuroprotection (91).

The effects of PROG are mediated by progesterone receptors (PRs), which are expressed throughout the CNS. These receptors play a pivotal role in the brain’s reward circuitry and modulation of dopamine dependent sexual behaviors, implicating the psychosocial impacts of reproductive hormones (92, 93). PROG also exerts neuroprotective effects, reducing oxidative damage in the CNS and supporting neuronal strength and plasticity, indicating its importance in the regulation of epilepsy (94, 95).

PROG is widely recognized for its anticonvulsant properties, primarily through its enhancement of GABA-A receptor-mediated chloride conductance causing neuronal hyperpolarization and reduced excitability (96–98). In addition to potentiating GABAergic inhibition, PROG also diminishes acetylcholine activity as well as amplifies the effects of adenosine, a neuromodulator with potent inhibitory effects. These mechanisms are relevant in syndromes such as autosomal dominant nocturnal frontal lobe epilepsy (99, 100).

A critical mediator of PROG’s anticonvulsant action is allopregnanolone (ALLO), a neuroactive metabolite that acts as a positive allosteric modulator of GABA-A receptors. ALLO concentrations rise significantly following PROG treatment and have been inversely correlated with seizure frequency due to the augmentation of GABA-A receptors, especially in women experiencing premenstrual seizure exacerbations (106, 107). Conversely, reduced ALLO levels in the cerebrospinal fluid have been associated with catamenial epilepsy and menstrual migraine, highlighting the clinical relevance of neurosteroid fluctuations in seizure susceptibility (108).

Clinical evidence supports the anticonvulsant effects of PROG in both focal and generalized epilepsies. Several studies have demonstrated reductions in interictal epileptiform discharges and seizure frequency following intermittent PROG administration, particularly in women with catamenial epilepsy (48, 101–103). However, the effectiveness of PROG appears to be dose-dependent, with insufficient levels showing minimal benefit (104, 105).

## Testosterone

Testosterone is the dominant male androgen and a steroid sex hormone integral to both reproductive and systemic health. Synthesized primarily in the Leydig cells of the testes under LH stimulation, testosterone is also produced in smaller quantities by the adrenal glands, ovaries, and placenta in both sexes. In the body, testosterone can exist in several main forms, the majority being inactive and bound to sex hormone binding globulin (SHBG), and a small active portion found unbound or weakly bound to albumin. The most biologically active form, 5a-dihydrotestosterone (5a-DHT) converted from testosterone via 5a-reductase, is vital to the development and functioning of primary and secondary male sexual characteristics. 5a-DHT exerts effects through both cytoplasmic and nuclear androgen receptors resulting in development of male secondary sexual characteristics, maintenance of spermatogenesis, muscle growth, libido, and mood regulation. While this hormone is also found in females to a much lower extent, excessive androgen levels can contribute to masculinization and hirsutism (46, 109).

Beyond its role in sexual development, testosterone contributes significantly to psychological well-being due to its role in mood regulation, self-esteem, and perceived quality of life (QOL), particularly in aging men. Conversely, testosterone deficiency is linked to sexual dysfunction, fatigue, and diminished QOL, all of which can extend the burden of epilepsy (109).

Emerging epidemiological evidence indicates a substantial link between androgen-related disorders and epilepsy in men. Both factors are implicated in a bidirectional relationship due to mechanisms causing a feedback influence via the hormones metabolic pathways. Testosterone can be aromatized to E2, which is mostly associated with proconvulsant properties, or metabolized by 5α-reductase into 5α-DHT, which exhibits anticonvulsant effects (110). Since 5a-DHT is the principal circulating male androgen, which is known to be an anticonvulsant, it can be estimated that there is a positive net impact of testosterone on seizure activity. However, MWE are known to have hypogonadism, characterized by reduced levels of free testosterone and its urinary metabolites, thus diminishing any potential anticonvulsant effects. Temporal lobe seizures (TLS) can disrupt the HPG axis, impairing gonadotropin secretion and subsequently reducing testosterone synthesis resulting in proconvulsant states. Furthermore, serum testosterone levels are affected by hormone-binding protein concentrations, which enzyme-inducing ASMs can alter. Newer ASMs appear to have a lesser effect on this mechanism (111). These hormonal imbalances have remarkable clinical implications for both sexes, albeit with differing outcomes.

The effects of testosterone on mood and reproductive function are complex in WWE. A recent study correlating elevated levels of testosterone, lower prolactin concentrations and increased depression scores connects any associations between the imbalance of androgens and their negative effects on mood and sexual desire (112). In MWE, hormonal therapies may offer dual benefits for both seizure control and sexual health. A comparative study evaluating the effects of anastrozole plus testosterone versus placebo plus testosterone for the treatment of sexual dysfunction and hypogonadism demonstrated a reduction in seizure frequency in both treatment arms (113). In this study, forty men with focal epilepsy, hyposexuality and hypogonadism were randomized into two groups and observed for three months. The improvement of seizure control was remarkably associated with decreased serum E2 levels and lowered scores in Beck Depression Inventory-II (BDI-II). Such an observation indicates a potential link between estrogen suppression, mood stabilization, and induced libido.

## Prolactin

Prolactin (PRL), a polypeptide hormone secreted by the anterior pituitary gland regulates lactation, reproductive physiology and immune modulation. It is also known to affect the central nervous system and can influence mood, emotions and behavior. Its pulsatile secretion pattern plays an essential role in homeostasis and reproductive balance and any alterations to this pulsatility often seen in neurological disorders such as epilepsy can trigger menstrual irregularities, infertility, as well as metabolic and immunologic imbalances (114).

Physiologically, PRL exhibits a well-defined circadian rhythm often mimicking the patterns of melatonin. Serum PRL levels surge during the onset of sleep and often return to baseline levels within one to two hours of waking. Baseline PRL concentrations are typically higher in women than in men, reflecting differences in endocrine regulation and reproductive physiology (116, 122). Stressors including pregnancy, lactation and trauma can also result in increased PRL levels (115, 116). The regulation of PRL secretion is primarily governed by the ventromedial and arcuate nuclei of the hypothalamus, which exert consistent inhibitory influence over the anterior pituitary through dopaminergic signaling pathways (115, 116). Disruptions in these inhibitory mechanisms, such as those caused by epileptic discharges can lead to transient elevations in serum PRL levels causing activation of many anterior pituitary hormones. The elevations in the hypothalamus receive input from key limbic regions, particularly the hippocampus and amygdala, structures frequently affected by epilepsy. These regions also play key influential roles on the dynamics of PRL dynamics through distinct anatomical pathways: hippocampal projections via the fornix and stria terminalis are predominantly inhibitory, while amygdaloid efferents through the amygdalofugal tract often exert excitatory effects (117–121).

In TLE where seizure activity typically originates in the mesial temporal structures, dissociation of epileptiform discharges from the temporal lobe to the hypothalamus may result in a post-ictal surge in PRL levels, as observed in both clinical and experimental settings (118, 120, 122, 123). This immediate surge in PRL levels, known as postictal hyperprolactinemia, serves as an indicator for generalized or focal seizures involving the limbic system. Elevations in hormone levels caused by ictal episodes are generally more pronounced in intensity and duration compared to those associated with stress, allowing PRL to be a reliable biomarker for seizures (115, 116, 124).

Increased PRL levels following seizures and during the interictal period may play a role in anovulatory cycles by interfering with other regulatory hormones secreted by the hypothalamus and anterior pituitary, in WWE. Elevated PRL concentrations have been observed after both electroconvulsive therapy and spontaneous seizures and studies have consistently shown that PRL levels are higher in individuals with focal epilepsy compared to their healthy counterparts (125). This increase in PRL has been documented in a significant proportion of generalized tonic–clonic seizures (GTCS) as well as focal seizures with secondary generalization (21, 126). Furthermore, clinical observations suggest that interictal brain activity may contribute to the sustained elevation of PRL levels in the blood, providing additional insight into its potential impact on reproductive health in WWE (127).

## Oxytocin

Oxytocin (OT), a peptide hormone produced in the supraoptic and paraventricular nucleus of the hypothalamus and secreted from the posterior pituitary, plays a critical role in the female reproductive system regulation, mainly during labor and lactation. It facilitates the milk ejection reflex, uterine contractions and constricts blood vessels after child-birth to prevent excessive bleeding (128). Beyond its effects on reproduction, OT influences a range of physiological and psychological processes, including social behavior, anxiety, appetite regulation, memory, learning, pain modulation (antinociception), social recognition, and the stress response by functioning as a neurotransmitter and neuromodulator by activating central OT receptors in the brain (129).

Both OT and vasopressin mRNA expressions are upregulated in the paraventricular hypothalamic nucleus during seizures (131). This suggests that the rapid rise in OT levels during or immediately following a seizure may represent a neuroprotective mechanism aimed at regulating hypothalamic activity in response to seizure-related stress (132, 133). For example, after KA-induced status epilepticus, increased activation of OT-expressing magnocellular neurons, but not vasopressin-expressing neurons, was observed in the PVN (133). Although OT and vasopressin share structural similarities, they likely serve distinct roles in the pathophysiology of epilepsy.

Due to the potential neuroprotective role of oxytocin following seizures, specific reports suggest that OT may have a therapeutic effect in neuropsychiatric disorders, including but not limited to epilepsy (131, 130).

## Discussion

The relation between epilepsy, reproductive hormones, the HPG axis and their complex pathways has been long recognized and studied, with plenty of documentation supporting the bidirectional influence between seizures and hormones. The interactions between seizures and the mechanisms of the HPG axis has a pivotal role in the functioning of the reproductive system thereby influencing fertility, libido and overall QOL. Estrogen is primarily proconvulsant enhancing glutamatergic excitation and reducing GABAergic inhibition, especially with exogenous estrogens associated with hormonal replacement therapy (HRT) or in-vitro fertilization (IVF). Seizures can disrupt GnRH/FSH pulsatility disrupting estrogen and progesterone levels thereby producing cycle irregularity and anovulation, however, utilization of HRT or exogenous hormones to treat any cycle irregularities can further exacerbate seizures. ALLO enhances GABA-A currents behaving as an anticonvulsant in the setting of seizures, however, epilepsy reflects low progesterone levels and are found to be associated with an increased estrogen to progesterone ratio. A luteal phase deficiency marked by lower levels of progesterone may result in difficulty for the fetus to implant and remain in the uterus (134). Hormonal fluctuations directly influence fertility outcomes such that luteal insufficiency and hyperprolactinemia contribute to anovulation and hypogonadism, while hyperandrogenic states, including polycystic ovary syndrome, are more prevalent in women with epilepsy. Conversely, reproductive hormonal therapies can exacerbate seizure risk, as demonstrated by case reports of seizure worsening during estrogen-based ovulation induction.

Much of the current documentation regarding this relation stems from animal based studies conducted in the 1990s, posing a translational challenge to humans due to the vast difference in epileptic properties and manifestations between both groups. These properties may depend on various factors such as on sex, treatment duration, time from seizure to testing, method of administration, hormonal status, brain region involved, and seizure type. According to the revised International League against Epilepsy (ILAE), the updated classification of seizures has removed the term ‘onset’ from ‘generalized-onset seizures’, acknowledging evidence that even generalized seizures may have focal origins. Essentially, this challenges any previous assumptions about the uniformity of seizures or hormonal responses to seizures as their focal nature may change. This nuance is critical when studying the pro or anticonvulsant properties of hormones, which may vary by seizure onset type (78, 79, 135). Importantly, the type and localization of seizures dictate the extent to which endocrine and reproductive hormones can be altered. Among focal epilepsies, temporal lobe epilepsy is recorded to have a significant impact on GnRH pulsatility by causing disruptions to the HPG axis, where right sided temporal foci are associated with hypogonadotropic hypogonadism causing reduced gonadotropin secretion, while left-sided temporal foci are associated with hyperandrogenic and PCOS causing increased LH to FSH ratios and chronic anovulation (48, 49). In contrast, generalized clonic–tonic seizures are associated with elevations in neuroendocrine hormones such as prolactin and cortisol which can also influence fertility outcomes which contribute to anovulation and hypogonadism (116, 115). The postictal rises in hormonal levels are dependent on the hormone, where prolactin is detected and measured 10–20 min after an epileptic episode, returning to baseline in 2–6 h (117–121). Due to this, prolactin serves as both a diagnostic biomarker and a marker of acute hypothalamic–pituitary stress activation in the setting of epilepsy. In terms of chronic reproductive dysfunction caused by epilepsy, a thorough endocrine evaluation comprising measurements of gonadotropins, estrogen, luteal phase progesterone, early morning testosterone in men, prolactin and cortisol are necessary to highlight the complex interplay of hormones that rise to reproductive dysfunction. Taken together, reproductive dysfunction in epilepsy emerges not from a single factor but from a convergence of seizure-related hypothalamic disruption, acute postictal hormonal surges, chronic alterations in HPGl feedback, and iatrogenic drug effects. Recognition of these multidirectional pathways highlights the importance of both acute and longitudinal endocrine monitoring, tailored by seizure type and patient sex, especially in individuals with fertility aspirations.

The new framework also shifts seizure observations which were previously categorized as motor and non-motor responses, to observable vs. unobservable manifestations. In essence, small changes as facial flushing or tachycardia which were previously overlooked phenomena can now be acknowledged as such as stress-induced sympathetic activation and be systematically linked to postictal endocrine disruptions (135).

The current understanding of epilepsy’s effects on hormonal balance relies predominantly on animal studies, with limited data regarding the impacts on humans. This poses a concern toward the validity of these studies as the metabolic demands, hormonal regulation and even seizure manifestation differs significantly between animals and humans. Currently, many of the studies regarding reproductive hormones and their role versatility in epileptic states are based on rodent models, which experience estrous cycles that last 4–5 days, with a considerable overlap between estrogen and PROG levels. This contrasts with the human menstrual cycle, which spans 28–35 days and features more distinct hormonal phases (136). Animal based studies have also indicated that PROG and its active metabolite, ALLO, both possess anticonvulsant properties and can lead to decreased seizure occurrence (106, 103), however, a 2012 clinical trial found that cyclic PROG was ineffective in treating partial refractory epilepsy in women (18). This clinical trial also paved the understanding regarding the differences in types of seizures and exacerbations and how they can manifest with various responses to hormones or treatments. It was noted that women with higher perimenstrual seizure exacerbations showed minimal response to PROG, but those with lower exacerbations showed some response to treatment., highlighting the inconsistencies of extrapolating animal data to human treatment and the need for further clinical research. Moreover, such discrepancies suggest the need to examine the hormonal responsiveness in relation to seizure type and timing within the menstrual cycle. The effects of medications observed around periods of menstruation further raise questions regarding the role of fluctuating hormones in seizure exacerbation and treatment efficacy. Much of the current literature focuses on acute postictal hormone measurements, often overlooking the physiological, chronic fluctuations of hormones, as the hormonal cycles do not follow a daily pattern but are subject to varying amplitudes based on the cyclic period. Studies by Bauer et al. (126) and Molaie et al. (127) identified acute PRL spikes following seizures and altered levels during interictal periods, suggesting persistent hormonal disruptions in epilepsy, though the long-term impact on seizure thresholds or fertility remains unclear. This constrains our ability to draw precise correlations and conclusions applicable to human healthcare.

Catamenial epilepsy, where seizures cluster around specific menstrual phases, highlights the need for longitudinal hormone monitoring, yet most studies measure hormones only postictally or within narrow windows, neglecting the full cyclical variation across the menstrual cycle (18). Consequently, the broader relationship between seizure patterns and chronic effects of hormonal fluctuations remain poorly understood (37, 62). Hormones naturally fluctuate in response to both extrinsic and intrinsic stimuli, and the inability to account for these variations weakens claims about their relationship to epilepsy. While acute postictal hormone changes are well established, long-term hormonal imbalances require longitudinal investigation.

Although hormonal influences in women have been more extensively studied due to the clear cyclic patterns, hormones like testosterone and PRL also fluctuate in men and may modulate seizure activity, particularly in conditions like stress or sleep deprivation. Epilepsy manifests differently between sexes, not only in terms of seizure frequency or type, but also in terms of drug response, psychiatric comorbidities, and endocrine profiles. For instance, lower testosterone levels were associated with increased seizure susceptibility, and hormone replacement therapy with testosterone has shown potential in reducing seizure outcomes. However, the precise role of testosterone remains unclear due to its dual metabolic mechanism in the brain, underscoring the need for further investigation of hormonal fluctuations in men.

In addition to the methodological limitations of conducting human studies, the lack of sex-specific analyses, longitudinal studies, and inadequate translation from animal research to human studies hinder our understanding of the role of epilepsy on hormonal imbalance. The physiological variations of hormones, the heterogenic manifestation of epilepsy, and sample size limitations pose significant challenges in conducting human research and performing analyses. Given the variability in reproductive hormones across age groups and menstrual cycles, along with diverse epilepsy phenotypes, large, meticulously stratified cohorts are essential to account for the diversity and identify clinically meaningful patterns.

Future research should aim to address the bidirectional relationship between seizures and the reproductive endocrine system through more precise, longitudinal studies. Careful monitoring of hormonal measurements in respect to seizure onset, menstrual cycle phase, and ASM exposure would allow for stronger comparisons across cohorts, especially with human studies and long term analyses. Advances in hormonal monitoring and imaging of hypothalamic–pituitary activity could refine our understanding of how seizure localization affects endocrine function. Personalized hormonal therapies, such as progesterone supplementation for catamenial epilepsy or androgen supplementation in men with hypogonadism to mitigate seizures and increase fertility outcomes could represent promising avenues to address issues regarding epilepsy and fertility. Advancements in this field could yield a deeper understanding of these mechanisms that will not only refine treatment strategies but also enhance the QOL for patients confronting difficulties of epilepsy and hormonal dysfunction.
